# *‘We’re here to listen and help them as well’*: a qualitative study of staff and Indigenous patient perceptions about participating in social and emotional wellbeing research at primary healthcare services

**DOI:** 10.1186/s12888-019-2263-8

**Published:** 2019-10-07

**Authors:** Sara Farnbach, Graham Gee, Anne-Marie Eades, John Robert Evans, Jamie Fernando, Belinda Hammond, Matty Simms, Karrina DeMasi, Maree L. Hackett, on behalf of the Getting it Right Investigators, Maree Hackett, Armando Teixeira-Pinto, Nick Glozier, Timothy Skinner, Deborah Askew, Graham Gee, Alan Cass, Alex Brown

**Affiliations:** 1 The George Institute for Global Health, PO Box M201, Missenden Road, Camperdown, NSW 2050 Australia; 2 University of New South Wales, Sydney, 2052 Australia; 3 The University of Sydney, Sydney, NSW 2006 Australia; 4 Victorian Aboriginal Health Service, 186 Nicholson St, 3065, Fitzroy, VIC 3072 Australia; 5 University of Melbourne, Melbourne, VIC 3000 Australia; 6 The University of Technology, 15 Broadway, Ultimo, NSW 2007 Australia; 7 The Glen Centre (Ngampie), 50 Church Rd, Chittaway, NSW 2261 Australia; 8 Nunkuwarrin Yunti of South Australia, Adelaide, South Australia 5000 Australia; 9 Aboriginal Medical Services Alliance Northern Territory, Moonta House 43 Mitchell Street, Darwin City, Northern Territory 0801 Australia; 10 The University of Central Lancashire, Preston, PR1 2HE UK

**Keywords:** Depression screening, Primary healthcare, Aboriginal and Torres Strait Islander, Qualitative research

## Abstract

**Background:**

Research can inform culturally-appropriate care to strengthen social and emotional wellbeing (SEWB) among Aboriginal and Torres Strait Islander (hereafter, the term ‘Indigenous Peoples’ is respectfully used and refers to all Aboriginal and/or Torres Strait Islander Peoples of Australia). We acknowledge the cultural diversity of Australia’s Indigenous First Peoples and they do not represent a homogenous group.) (hereafter Indigenous) Peoples. We explore the perspectives of primary healthcare staff and Indigenous patients about their willingness to and experiences participating in SEWB research.

**Method:**

Process evaluation using grounded theory approaches of *Getting it Right*: *The validation study*, a national validation designed Indigenous SEWB research project (*N* = 500). Primary healthcare staff (*n* = 36) and community members (*n* = 4) from nine of ten primary healthcare services involved with the research project completed qualitative semi-structured interviews. Interview data were triangulated with participant feedback (responses to structured questions and free-text feedback collected during *Getting it Right*), study administrative data (participant screening logs, communication logs, study protocol, deviation logs and ethics correspondence) and interviewer field notes.

**Results:**

Three themes about staff, patient and community perspectives concerning research participation developed: (1) considering the needs, risk, preferences and impact of participation in research for staff, patients and community; (2) building staff confidence speaking to patients about research and SEWB problems and (3) patients speaking openly about their SEWB. Some staff described pressure to ensure patients had a positive experience with the research, to respond appropriately if patients became upset or SEWB problems were identified during interviews, or due to their dual role as community member and researcher. Patients and staff reported that patients were more likely to participate if they knew the staff outside of the service, especially staff with a shared cultural background, and they perceived SEWB as a community priority. Staff reported their skills speaking to patients about the research and SEWB improved during the research, which built their confidence. Contrary to staff preconceptions, staff and patients reported that many patients appreciated the opportunity to speak about their SEWB and contributing to research that may eventually enhance SEWB in their community.

**Conclusion:**

Our research project was considered acceptable by most staff and patients. The positive outcomes reported by staff and feedback from patients highlights the importance of providing opportunities for people to speak about their SEWB and for research-informed SEWB PHC care.

**Trial registration:**

Getting it Right is registered on ANZCTR12614000705684.

## Background

Research focused on Aboriginal and Torres Strait Islander (hereafter referred to as Indigenous^i^) people may inform evidence-based and culturally-appropriate strategies that strengthen social and emotional wellbeing (SEWB) [1]. When planning and conducting this research, consideration of its impact on participants, research staff and the community is important to ensure it leads to joint ownership, tangible benefits in participating communities and is feasible.

While strengthening SEWB by building resilience is one focus of many Indigenous-focused PHC services [2, 3], delivering care [4] and conducting research [5] focused on the assessment and treatment of mental health problems may also be needed to reduce the high rates of psychological distress experienced by Indigenous Australians compared to non-Indigenous Australians [6].

When considering whether to become involved with SEWB research, PHC staff likely consider their experiences with earlier research projects, general preconceptions about research and the topic (whether grounded in experience or not). Negative experiences such as involvement with research perceived as resulting in little or no tangible benefit to the community [7], or limited to describing the size and nature of the problem, without offering solutions [8], or concerns that asking about suicidal ideation may increase suicidal tendencies, may deter staff from becoming involved [9]. PHC staff and patients perspectives should be central during research planning to ensure it provides tangible benefit, is relevant, effective, culturally respectful and feasible [10, 11].

We present the results from a process evaluation designed to explore the perspectives of PHC staff and Indigenous patients about their willingness to and experiences of participating in research and speaking about SEWB. The work was part of a NHMRC-funded, national Indigenous-focused SEWB PHC-based research project *Getting it Right*: *The validation study* [12] (hereafter the research project), conducted in ten PHC services (hereafter participating services).

## Methods

The methods of the research project and process evaluation have been previously described [12, 13]. In brief, coordinating staff at participating services invited staff and community members (purposive identification [14]) to complete qualitative semi-structured grounded theory [15] interviews. Staff interviews were conducted by SF between November 2016 and June 2017 (after recruitment was completed for the research project) in a confidential setting, in-person at the participating service or over the phone. SF is a female registered nurse and PhD candidate, and has completed training in qualitative data collection, analysis and reporting. She was project manager of the research project and had relationships with staff and community members for between 1 and 3 years. All interviews and most of the thematic analysis were completed before the results of the research project were released to SF, the Indigenous Advisory Group (GG, JE, AME, JF, MS, BH and KD) or participating communities.

The research project [12] was designed to determine the validity of a culturally-adapted depression screening tool (the adapted-Patient Health Questionnaire-9) [16] for use by Indigenous people and recruited 500 participants (2014 to 2016). It was managed centrally from The George Institute for Global Health in Sydney, Australia. The study protocol [12] was adaptive, meaning participating services nominated existing or hired new staff to conduct the research (based on their assessment of staff skills, backgrounds and availability) and developed individualised recruitment and safety follow-up plans (with support from researchers) while the core elements of the protocol were unchanged. One staff member interviewed consenting participants (Inidgenous PHC patients) using the depression screening tool [16] and another using the semi-structured MINI International Neuropsychiatric Interview (MINI) 6.0.0 (depression, anxiety and Post-Traumatic Stress Disorder (PTSD) modules) [17]. Patient interviews involved questions about SEWB problems (defined as depression, anxiety and PTSD, and thoughts of self-harm, suicidal ideation or intent) and feedback on the research.

We defined ‘patient’ as PHC patients in general or before they consent to participate in the research project and ‘participant’ as a patient who has provided informed consent. SEWB includes mental health within a holistic framework that recognises wellbeing as interconnected with land, culture, family and community and recognises the role of historical, political and cultural determinants [18]. Indigenous-focused PHC services include Aboriginal Medical Services and Aboriginal Community Controlled Health Services.

Process evaluation staff interviews were conducted using interview guides, digitally recorded and transcribed verbatim. NVivo 10 for Windows [19] was used to manage the data. Qualitative interview data were triangulated with participant feedback about the depression screening tool and their experiences with the interview (responses to questions and free-text feedback entered onto case report forms by patients or interview staff immediately after the first research interview), administrative data from the research project (participant screening logs, communication logs, study protocol and ethics correspondence) and field notes (SF). To identify if there were groups of staff who more commonly reported specific or divergent views, we considered the developing themes according to participant characteristics (gender, ethnicity, years working in the role). We also report our observations of the research project as project manager (SF) and investigators (GG and MH).

Process evaluation staff interviews continued until all potential staff or community members were considered by the coordinating staff. Data were coded inductively. No new open codes were identified in the final two interviews, indicating data saturation. Interviews and coding were conducted in three stages and codes constantly compared during analysis. Authors were provided with regular reports of interviews and emerging themes. A record of codes, their properties, our interpretations, and feedback from authors were kept in memos. Codes and memos were grouped into themes, which were integrated into subsequent interview guides (total = 3). SF and AME piloted interview guide one. Ten (25%) transcripts were independently double-coded by Aboriginal authors (GG and AME).

This process evaluation was conceived, designed and conducted while following the Values and Ethics Guideline [10] and received state-based ethics approval (refer to protocol) [13]. Consent from each participating service was also provided.

## Results

Interviews were completed with 36 staff (34 individually and as a group interview) and four community members (group interview) from nine of the ten participating services, resulting in 1324 min of transcribed interviews. Due to staff turnover and organisational change at the tenth service, these staff did not complete interviews. Managers (*n* = 10), Aboriginal Health Workers (AHW) (*n* = 9), Allied Health Staff (*n* = 8), Research Coordinators (*n* = 5), and General Practitioners (GPs) (*n* = 4) were interviewed (Table 1).

**Table 1:** Characteristics of staff and community members who completed qualitative interviews

Characteristics	*N* = 36
Staff characteristics	
*Gender*	
Female	24
*Ethnicity*	
Indigenous	17
*Years working at participating health services*	
Less than one year	0
1–2 years	11
2–3 years	2
3–4 years	6
5+ years	13
Data unavailable	4
Community members characteristics	*N = 4*
Female	2
Indigenous	4

Three themes related to staff, patients and community perspectives about their willingness to participate in SEWB research developed: (1) considering the needs, risk, preferences and impact of participation in research for staff, patients and community; (2) building staff confidence speaking to patients about research and SEWB problems and (3) patients speaking openly about their SEWB. Analysis of staff interview data according to participant characteristics did not identify divergent views between participants with different characteristics.

### Theme one: staff considering the needs, risks, preferences for and impact of participation in SEWB research for staff, patients and community

Staff said the research project was needed because it addressed SEWB, which was a community priority (Table 2). Staff described feeling pressure surrounding how patients would perceive or respond to the research project, which they managed by assessing if patients were suitable to participate, including considering patients’ personal circumstances and connection with staff, before inviting them to participate. Some staff (mostly managers) assessed which staff were suitable to conduct the research interviews, speak to patients about SEWB problems and provide follow-up referral (if required). Some staff described preparing themselves to hear about traumatic events during the research interviews.

**Table 2:** Theme one – staff considering the needs, risks, preferences for and impact of SEWB research participation for staff, patients and community

Subtheme	Description of subtheme
Perceiving a need	For research addressing community priorities
Feeling pressure	To ensure patients had a positive experience with the research, which could be harmed if: – Patients respond negatively to depression as a topic – Patients become upset from speaking about SEWB problems – Patients are offended by being asked about research/SEWB problems To respond appropriately to patients who became upset or if SEWB problems were identified during research interviews Because their dual role as researcher and community member contributed to pressure to ensure that research benefited patients and community after completion
Assessing suitability	Of patients’ circumstances before inviting them to participate Of skills of interviewing staff to assess and treat SEWB
Being prepared	To support patients appropriately (if needed) To ask about suicidal ideation/intent or hear about traumatic events

*Abbreviations*: *SEWB* Social and emotional wellbeing

#### Perceiving a need

Many staff reported that there was a need for SEWB research because it addressed a priority in their community or there was a lack of research to inform SEWB care:

We do need an appropriate screening tool to help pick up people with depression and suicidal ideas so we can manage those things specifically, effectively in primary care. (Non-Indigenous, GP, male, #9)

#### Feeling pressure

Many staff described pressure to ensure patients had a positive experience with the research project, and viewed participation as a potential risk because patients may have a negative response to the topic of depression, become upset during interviews or be offended by being asked to participate.

Some staff described depression as a ‘sensitive’ topic or suggested that patients may be concerned about stigma if diagnosed with depression. These staff perceived that patients’ perceptions could be a barrier for patients when considering research participation:

So me and the other research officer thought, hmmm, might be a bit of a tight one, people opening up about their inner feelings. A lot of blacks don’t like doing that. (Indigenous, AHW, female, #4)

Some staff reported initial concerns about speaking to patients about SEWB problems because it could cause problems for patients, by bringing up upsetting or traumatic issues. By asking patients to repeat traumatic stories during the research interviews, staff were concerned they may unnecessarily burden patients by *‘flaring things up,’* especially if an existing condition was known to clinicians and they were already receiving treatment (Non-Indigenous, GP, male, #9).

Some staff reported feeling concerned they could offend patients by inviting them to participate in research about depression. These staff described carefully framing the conversation to avoid:

Pigeonhole or tag [ing] people that have mental health conditions (Non-Indigenous, GP, male, #1).

Several staff reported feeling pressure to respond appropriately to patients if they became upset, were identified with a disorder (depression, anxiety or PTSD) and/or indicated thoughts of suicidal ideation or intent during a research interview. Some staff reported concerns that they may not be equipped to deal with these situations, while for other staff, this was not a concern because it was ‘*part of the job’*.

Several staff who were also involved with their local community reported that their dual role (researcher and community member) contributed to pressure to ensure that research had benefit to patients and community. One AHW described being *‘the face of the research*’:

There comes a responsibility that sits on my shoulders then as the face of that, being the Aboriginal worker and being from this community … to ensure that it’s successful and that things work well, and that people are happy with the way things go. (Indigenous, AHW, female, #7)

#### Assessing suitability

Several staff described assessing patients’ suitability by considering the patient’s circumstances and their connections with the patient in the community before inviting them to participate. Patients deemed unsuitable by staff were those with multiple complex health priorities, an acute illness or who were experiencing stressful events which staff felt should be the focus during their visit to the participating service.

Staff, particularly managers, considered which staff were suitable to complete research interviews, by reviewing staff skills assessing and treating SEWB problems. One manager reported that having staff with existing SEWB assessment and treatment skills ensured they were prepared to respond appropriately if a SEWB problem was identified.

Some staff reported having to *‘think on their feet’* to immediately assess and manage unexpected issues that arose during research interviews. For example, one staff member heard a story about:

Violence from their [participant’s] partner, who was the next room (Indigenous, manager female, interview 24).

#### Being prepared

Most staff reported prioritising planning the safety protocol [12] because it was important to support patients appropriately and minimise patients’ risks (by having a plan for follow-up care if required) and staff risks, by outlining a process if patients reported thoughts of self-harm or suicidal ideation or intent during a research interview.

Some staff also described preparing themselves emotionally to ask *‘tricky’* questions during the research interviews which sometimes involved *‘listening to traumatic stories’* (non-Indigenous, nurse, female, #22). Another described that hearing traumatic stories was difficult:

Family abuse, sexual abuse, domestic violence, children being taken away, and then the difficulty of getting a child back. I found that really, really difficult. And I remember one patient who just bawled their eyes out … And I ended up crying with her because the situation was so difficult. (Non-Indigenous, research coordinator, female, #6)

Some staff reported completing regular debriefing sessions and receiving support from managers around maintaining a work-life balance. While this was not reported by all staff, we did not specifically ask about it during the process evaluation.

### Theme one – patients considering needs, risks, preferences and impact of research participation for community and themselves

Patients appeared to prefer to participate in research and speak about SEWB problems if they were comfortable in the environment, perceived that the research addressed a community priority and/or had a connection with a staff member. According to staff, some patients choose not to participate because they had concerns about research, about speaking about depression or were too sick or busy (Table 3).

**Table 3:** Theme one – patients considering the needs, risks, preferences and impact of research participation for community and themselves

Subtheme	Explanation of subtheme
Feeling comfortable	In the physical environment/setting where research is occurring
Perceiving a need	For research addressing community priorities
Having a connection	Between staff and patients, including shared cultural background (contrasting perspectives explored in Table 4) Sometimes connections can: – Be inappropriate if interviewing family members – Require additional time to complete research interviews
Declining to participate	Because of concerns about research or speaking about SEWB problems Too busy, too sick or had other priorities

*Abbreviations*: *SEWB* Social and emotional wellbeing

#### Feeling comfortable

Some staff reported that patients were more likely to complete research interviews and speak about their SEWB outside the clinical environment where they were more relaxed, for example in a local park, car or in their own homes. At one participating service, all patients regularly participated in group counselling sessions and this was cited as a reason for the high recruitment rate of 100% because:

It may have been easier for these guys to talk about their emotional state (Indigenous, GP, male,# 35).

#### Perceiving a need

Some patients reported that SEWB was priority in their community and this research topic motivated them to participate:

[The research is] beneficial for Aboriginal people getting into depression. (Indigenous participant, female, 61 years)

Verifying this view, many staff reported that patients were interested in participating because of the research topic. According to one GP, patients were:

Impressed the service was doing something about it [depression] (Indigenous, GP, male, #35).

#### Having a connection

In participant feedback, 90% reported feeling comfortable participating and this may be due to their existing connection with the staff conducting the research interviews. In the free-text feedback one participant reported:

I felt comfortable answering the questions because I was talking with someone I trusted, if it was a stranger I would feel different. (Indigenous participant, male, 71 years)

Having a cultural connection also contributed to patients’ comfort. One AHW reported clarifying information during informed consent by *‘speaking the lingo’* (Indigenous, AHW, female, #4), rather than using the formal academic language on the consent form, which resulted in patients participating in the research. Participant feedback verified that a shared cultural background made them comfortable answering the questions:

Because a Murri woman from this community was asking them. (Indigenous participant, female, 56 years)

The perspectives of some staff and patients about the impact of their connections differed, with some staff reporting that they had concerns that their connections would dissuade patients from participating, and patients reporting this connection made them comfortable to participate (Table 4).

**Table 4:** Theme one – contrasting perspectives of staff and patients about having a connection

Explanation of having a connection	Staff perspective	Patients’ perspective
Between staff and patients, including shared cultural background	– Some staff perceived that patients may be concerned about confidentiality due to connections, and therefore may not participate or be willing to have SEWB discussions – Some staff were surprised that their connections encouraged patients to participate – Some staff perceived their connections with patients established trust, which facilitated participation	Patients reported connections made them comfortable to participate and have SEWB discussions (through established trust and/or shared cultural background)

*Abbreviations*: *SEWB* Social and emotional wellbeing

Staff perspectives about how their community connections would impact on patients’ willingness to participate were mixed. During start-up training and process evaluation interviews, some staff reported that some patients may be unwilling speak to staff who they knew from the community, because of concerns that their personal information may be shared:

I didn’t know how people were going to open up to me … they were either going to be more comfortable with me and happy to share or they were going to be no, I’m not going to say nothing because you know my family. (Indigenous, AHW, male, #5)

Despite initial concerns, once research interviews begun, these staff were surprised to realise that their role within the community encouraged patients to participate because it fostered patients’ trust with staff and therefore the research:

I thought they might not do it … But no, they were fine with me doing it actually. I think some of them did it because it was me. (Indigenous, AHW, female, #4)

In contrast, other staff reported their connections were important to establish trust which may increase the accuracy of data because *‘you don’t get the story unless you know the person*’ (Indigenous, manager, female, #24). One RN described a couple who were:

First asked by someone else [to participate] and they said no, but said yes to me because they knew me and had a relationship with me. (Non-indigenous, RN, female, #21)

Some staff described how these connections prolonged the research interviews. One AHW recalled feeling pressure to complete interviews in shorter timeframes because of other service priorities. This was difficult because when a patient is:

Opening up to you … you can’t just get what you want, okay, get out the door. It doesn’t work like that in our mob. We’re here not just for the research. We’re here to listen to them and help them as well. (Indigenous, AHW, female, #4)

#### Declining to participate

Staff reported that some patients did not participate because of concerns about research generally, speaking about their SEWB or they were too busy, sick or had other priorities. The screening logs showed a participation rate of 55% (number screened/number participated). Most of the patients who were screened and did not participate declined with no reason documented (64%) or were ineligible (32%) because they did not meet the inclusion/exclusion criteria.

Two AHWs reported that the community had concerns about research generally and one described *‘defend [ing] research’* when talking about the research with some patients (Indigenous, male, AHW, #10). These AHWs reported that these concerns arose from patients’ negative experiences with research or suspicion about the motives behind the research because the *‘government would check them out*’ (Indigenous, AHW, female, interview 28) if they participated.

In contrast to some patients who appeared to perceive that SEWB research was needed, some staff reported that some patients may be concerned about the stigma associated with depression and may have chosen not to participate. This stigma may have caused:

A reluctance of clients, they didn’t really want to talk about stuff when they realised it was about depression and anxiety. (Non-Indigenous, male, RN, #25)

### Theme two: staff building confidence speaking about research and SEWB problems

Staff became more confident speaking to patients about research and SEWB problems as they gained experience and skills conducting research interviews (Table 5).

**Table 5:** Theme two – building staff confidence speaking to patients about research and SEWB problems

Subtheme	Explanation of subtheme
Enhancing skills speaking about research and depression	From experience conducting research interviews From experience speaking to patients about SEWB problems
Enhancing staff-patient relationships	Through discussions arising from research
Perceiving positive outcomes	Through identifying problems and providing care Therapeutic benefit for patients from research interviews

*Abbreviations*: *SEWB* Social and emotional wellbeing

#### Staff enhancing skills speaking to patients about research

When reflecting on the research, some staff reported being nervous before the first research interview because they had minimal experience speaking to patients about SEWB or completing research. For some staff, their involvement sparked an ongoing interest in research. One GP went on to complete further study and reported that the skills of other staff were enhanced during the research (Indigenous, GP, male #35). Some staff reported that their experience with the research resulted in greater integration of conversations about SEWB into routine practice:

I was always a little bit reluctant to ask that stuff [SEWB assessment], whereas just now, for all workers, it’s become just sort of a normal part of the work process. (Aboriginal, AHW, male, #17)

Some staff described that the research empowered them in their work and personal lives, attributing this to their experiences speaking with patients about depression and responding appropriately:

I think that she [research particpant] left the interview feeling like a weight had been lifted off her shoulders … So you know, that was pretty empowering for me and it made me feel like well I’ve got a job to do here, you know. (Indigenous, AHW, male, #5)

#### Enhancing staff-patient relationships through research

Many staff reported that their relationships with patients were enhanced through conducting research interviews, because the interviews provided an opportunity for in-depth conversations about patients’ lives. These conversations built therapeutic relationships and developed connections *‘in a different way, on a different level’* (Indigenous, manager female, #24) to those had before the research project. During their usual non-reserach roles, oppotrunities for in-depth conversations were often limited because of time restraints. Staff reported that their enhanced relationships was a positive outcome of the research.

#### Perceiving positive outcomes

Most staff considered the many patients who were identified with depression, anxiety or PTSD during the research and provided with follow-up care was a positive outcome of the research:

Nine times out of 10 people were coming out with psychology appointments or psychiatrist appointments, or medication or both. (Non-Indigenous, RN, male, #3)

Staff reported that some patients become upset when talking about their problems and this concerned staff who responded by giving patients time to speak, offered support, provided referral (if necessary) and offered to stop the interview. Staff reported that sometimes more time was needed to provide follow-up care than what was originally allocated to the research, but that providing follow-up care was part of the job. These staff reported that upset patients wanted to continue interviews and the interviews may have had therapeutic benefit:

And I remember one patient who just bawled their eyes out and I tried to stop the interview, but she didn’t want to because she said she needed to get it out of her system. (Non-Indigenous, research coordinator, female, #6)

#### Counter-opinion of negative outcomes for participants

One staff member recalled a negative patient outcome from the interview:

The patient already had other mental health issues that their GP knew about anyway. And that patient talked about all that but also came up with some other things … At the end of it, I didn’t realise that that patient got a bit upset or distressed. (Non-Indigenous, research coordinator, female, #6)

### Theme three: patients speaking openly about SEWB

Many patients appeared to speak openly, share personal stories and appreciate the opportunity to participate, because it provided an opportunity to speak about their SEWB and to contribute to community outcomes. Many staff reported being surprised that patients spoke openly because staff expected they would be uncomfortable speaking about SEWB problems: ‘*especially given the sensitivity of the topic’* (Non-Indigenous, GP, male, #9) or if they knew the interviewing staff member (Table 6).

**Table 6:** Theme three: Patients speaking openly about SEWB

Subtheme	Explanation of subtheme
Sharing personal stories	Patients speaking openly about family histories and cultural exchange
Appreciating the opportunity	To speak about their SEWB and SEWB problems To contribute to community outcomes

*Abbreviations*: *SEWB* Social and emotional wellbeing

#### Sharing personal stories

Many staff reported feeling privileged hearing patients’ stories during interviews. However, sharing stories prolonged interview timeframes:

I felt very privileged to be sitting down with people and starting difficult conversations … and there was one client … I was there for three and a half hours … he was telling me about his family and his connections and the disconnected side of things, talked a lot about repossession … Showed me his family history book, and where he was from and all about his Country and all the things that he’d put in place for his family. (Indigenous, manager, female, #24)

For some Indigenous staff, sometimes cultural information was exchanged. One AHW recalled interviewing Elders in his community who wanted to have lengthy discussions with him. This AHW reported feeling obliged to continue discussions and give the Elders time to talk due to cultural protocols, but was also aware that other participants were waiting to complete interviews, so had to find a way to politely shorten conversations.

#### Appreciating the opportunity

Some patients appeared to appreciate the opportunity to speak about their SEWB during the research and provided positive feedback about the interview questions or about having an opportunity to reflect on their SEWB:

Questions are good at making you think about things you would not normally think about yourself – that it was good to make [you] aware of your emotions and identify if you have any problems. (Indigenous participant, male, 32 years)

Many staff verified this perspective and reported that many patients appreciated speaking about their SEWB and that staff were interested in their lives:

But I think clients quite enjoyed being asked a particular question [relating to suicidal ideation]. It gave them an opportunity to talk. (Indigenous, research coordinator, female, #30)

One manager reported being thanked by the family of a participant after a lengthy interview:

And two days later … he’d become very unwell and subsequently passed away. And so really the last person that had had this big conversation with him was me … it is very powerful, because the family members came to me, saying, you really, you were one of the last, and how wonderful [participating service] was for providing this extra service. And I thought, in actual fact, it’s part of the study. (Indigenous, manager female, #24)

Some staff reported that patients appreciated contributing to community outcomes through their involvement with the research and ‘*enjoyed being part of something’* (Indigenous, AHW, male, #33). One GP reported how:

Impressed some of the clients were, in regard to mental health issues in men being addressed or being researched into … they were quite proud to be involved with that. (Indigenous, GP, male, #35)

Quantitative feedback from participants demonstrated overwhelmingly that participants were comfortable answering the questions (91%) and perceived that the screening tool was easy to understand (87%). 86% reported that they were comfortable with how much personal information was asked. Approximately 20% of participants provided free-text feedback, which mostly related to the depression screening tool (pertaining to specific questions and response category options) or to their positive experience with the research project:

Happy to take part in [the] study, through [sic thought] it was good that the research was being done (Indigenous participant, female, 40 years).

## Discussion

Overall, our results show that despite some initial uncertainty among staff, many patients were willing and appreciated participating in SEWB research, especially when they had existing connections with staff and perceived that the research addresses a community priority. Some staff reported that their confidence speaking to patients about SEWB improved and that some patients benefited therapeutically from participation, demonstrating potential ongoing positive implications of research. Some staff reported pressure from their dual roles within the community, highlighting a need to consider the wider implications of research for staff and patients and for flexible research protocols. These results illustrate some of the principles described by Jamieson et al. [11] about conducting beneficial, relevant, effective and culturally respectful research, and ensuring research addresses community priorities and incorporates capacity building.

Many staff and patients reported that building SEWB was a community priority [11]. The positive participant feedback demonstrated engagement with the research topic. This was a surprise to some staff who were initially concerned that patients may respond negatively when asked to participate in research about depression. Previous research shows that asking about suicidal ideation can reduce rather than increase suicidal ideation [9]. Some participants of trauma-related research viewed participation as a positive experience, regardless of their trauma history [20] and even those who became upset did not regret participation because they believed it had personal and community level benefit [21]. It is not surprising that staff reported that some patients seemed to benefit just from participation in the research project, regardless of whether SEWB problems were identified that would otherwise have been missed. This demonstrates that it is important to provide patients with opportunities to speak about SEWB and that trained staff can ask directly about SEWB problems.

Developing existing capacity is particularity relevant during Indigenous-focused research because local staff may have existing relationships within communities that may put some Indigenous people at ease, potentially facilitating interest in research. Previous research has indicated that some Indigenous people prefer speaking to staff who they have close and ongoing relationships with during research [22] and when accessing health care [23, 24]. In our research, staff-patient relationships appeared to facilitate participation and SEWB conversations, and some staff suggested it may also have improved the accuracy of the research data [25]. Involving Indigenous researchers may also enhance research through the local expertise they bring which may help to promptly identify participants [26], facilitate research that is in-line with cultural protocols, is respectful and addresses community priorities [27].

However, our research highlights some considerations for researchers working within their own community. Our protocol focused on the safety of patients and we assumed that staff care was provided by participating services as part of their usual processes. Pressure on staff, and staff preparing to hear traumatic stories, were raised as sub-themes in our research and we feel it prudent to remind researchers that the safety of all participants (community, patients and staff) is paramount. The role of AHWs as emotional brokers may contribute to emotional exhaustion leading to burnout [28], and the emotional labour resulting from cultural and family obligations, the complex needs of many clients or backlash if poor outcomes occur has been identified among Indigenous maternal health workers [29]. Similarly, an Indigenous researcher has highlighted the potential impact of their research on their relationships with other community members, the way they are viewed within their community or the way they viewed themselves [30]. Our research highlights the need to focus on the wellbeing of research staff during research.

To the best of our knowledge, this is the first Australian PHC-based, Indigenous-focused research exploring staff and patients’ perspectives around participating in research and speaking about SEWB. Our results suggest that when appropriately planned and supported, these services are a viable setting for SEWB research.

Our research confirms some of the principles described by Jamieson et al. [11] pertaining to the importance of research that addresses a community determined priority and is focused on enhancing capacity. Additionally, with use of an adaptive protocol, research can be flexible so staff can determine localised research processes while maintaining scientific rigour [11].

Delivering training to PHC staff about culturally-appropriate SEWB screening, assessment and treatment may enhance the likelihood of staff speaking to patients about SEWB outside of research. To ensure appropriate SEWB care is available at PHC services, referral pathways and evidence-based management guidelines are needed.

PHC services are well positioned to engage in SEWB research. When developing SEWB research, we recommend:

1. Identifying adequately trained, culturally-competent staff, or ensuring adequate training and support of staff is provided by the researchers,
2. Allocating adequate time for conversations around research and ensuring PHC services have capacity to follow up (if required) people who are identified with SEWB concerns,
3. Developing evidence-based SEWB management guidelines and referral options.

Finally, we suggest that the potential risks and pressures on Indigenous staff who participate in SEWB research may be minimised by ensuring staff have autonomy to manage cultural pressures, complete self-care and opportunities to access therapy or support.

### Strengths and limitations of this research

SF was the project manager of the research project and led the process evaluation allowing for in-depth understanding of the project and surrounding events which enhanced data collection, analysis and interpretation. The relationships and rapport developed during the research project facilitated discussions during staff interviews. However, these relationships may have influenced the process evaluation interviews because staff may have avoided reporting negative experiences related to the research project. By blinding the authors to the results of the research project, we reduced the risk of main study findings influencing the interview discussions.

Our ability to draw conclusions based on patients’ perspectives is limited to feedback collected during the research project after the first research interview and staff opinions of patients’ perspectives. The opinions of staff and patients unwilling or unable to engage with the research were not collected, potentially limiting us to not capturing the perspectives of the most unwell patients or specific reasons for non-participation among staff or patients. We are aware of at least two staff who were trained in the research and chose not to conduct reserach or process evaluation interviews.

Although staff from nine diverse PHC services contributed data, findings may not be generalisable to other Indigenous-focused PHC services. However, these data provide useful insights for future Indigenous-focused PHC SEWB research.

## Conclusions

This research project was considered acceptable by staff and patients who took part. The confidence of many staff speaking to patients about research and SEWB improved, and many patients were willing to and appreciated the opportunity to participate in the research project. These positive outcomes reported by staff and patients arising from participating in research highlight the importance of providing opportunities for people to speak about their SEWB and for research-informed SEWB PHC care. Together, these results indicate that when adequately planned and supported, research can have benefit beyond the research project.

## Data Availability

The datasets generated and analysed during the current study are not publicly available to preserve the privacy of participants.

## Abbreviations

AHW: Aboriginal Health Workers
GP: General Practitioners
PHC: Primary healthcare
PTSD: Post-traumatic stress disorder
SEWB: Social and emotional wellbeing
